# Effects of Training Interventions to Treat Postpartum Urinary Incontinence: A Meta‐Analysis

**DOI:** 10.1111/1471-0528.70014

**Published:** 2025-09-24

**Authors:** Cristina Gallego‐Gómez, Sergio Núñez de Arenas‐Arroyo, Ana Torres‐Costoso, Eva Rodríguez‐Gutiérrez, Vicente Martínez‐Vizcaíno, Sandra Martínez‐Bustelo, Claudia Andrea Quezada‐Bascuñán, Julián Ángel Basco‐López, Asunción Ferri‐Morales

**Affiliations:** ^1^ Faculty of Physiotherapy and Nursing University of Castilla‐La Mancha Toledo Spain; ^2^ Physiotherapy Unit, Health Center of Camarena Toledo Spain; ^3^ Health and Social Research Center University of Castilla‐La Mancha Cuenca Spain; ^4^ Research Network on Chronicity, Primary Care and Health Promotion (RICAPPS) Cuenca Spain; ^5^ Facultad de Ciencias de la Salud Universidad Autónoma de Chile Talca Chile; ^6^ Psychosocial Intervention and Functional Rehabilitation Research Group, Faculty of Physiotherapy University of A Coruña A Coruña Spain; ^7^ Research Group in Pediatric and Neurologic Physiotherapy, ImproveLab University of Castilla La Mancha Toledo Spain

**Keywords:** abdominal muscles, biofeedback, electric stimulation therapy, exercise therapy, parturition, pelvic floor disorders, physical therapy modalities

## Abstract

**Background:**

Urinary incontinence (UI) is a common symptom after childbirth. Training interventions are recommended for its management.

**Objectives:**

To evaluate the effects of abdominal and/or pelvic floor muscle training (PFMT) combined with other conservative tools.

**Search Strategy:**

The MEDLINE, Scopus, Cochrane Library, Web of Science and Physiotherapy Evidence Database (PEDro) databases were searched from inception to November 6th, 2024.

**Selection Criteria:**

Three reviewers independently reviewed titles, abstracts, and full texts.

**Data Collection and Analysis:**

Experimental studies addressing the effects of training interventions on UI severity during the postpartum period were included. The Hartung–Knapp–Sidik–Jonkman method was used to calculate pooled estimates of the standardised mean differences (SMDs) and their respective 95% confidence intervals (CIs). Subgroup analyses and meta‐regression models were performed according to population characteristics, intervention characteristics, and type of outcome measure. The protocol was registered on the International Prospective Register of Systematic Reviews (PROSPERO: CRD42023489312).

**Main Results:**

Nineteen published studies were included. There was no statistically significant difference in UI severity in the analyses comparing training interventions versus controls or education interventions (SMD = −1.08; 95% CI: −2.24 to 0.08). According to the pre–post analyses, PFMT (SMD = −1.45; 95% CI: −2.61 to −0.28), PFMT through electrical stimulation (ES)/biofeedback (BFB) (SMD = −2.16; 95% CI: −3.50 to −0.81), and PFMT combined with abdominal muscle training (AMT) (SMD = −1.73; 95% CI: −3.42 to −0.03) modalities showed a statistically significant reduction of UI in postpartum women.

**Conclusions:**

This meta‐analysis provides an overview of the evidence supporting PFMT alone or in combination with ES, BFB, or AMT as suitable conservative approaches for the treatment of UI in the postpartum period. Further studies are needed to establish recommendations for abdominal wall training alone in the treatment of UI.

## 1 Introduction

Urinary incontinence (UI) is the most common symptom of pelvic floor dysfunction in women worldwide [1, 2] and may have a negative impact on their physical, social, economic and psychological well‐being, thus affecting their quality of life [3]. Pregnancy and childbirth are considered to be among the most important risk factors for the onset and development of UI [4, 5] with an estimated prevalence during the first year postpartum ranging from 15% to 30% [6]. Postpartum UI is associated with biomechanical and hormonal changes that occur during the obstetric phase, such as changes in muscle and connective tissue, and those resulting from perineal trauma during delivery [7, 8].

Physiotherapy is the main option for UI [9], with pelvic floor muscle training (PFMT) being the most commonly used technique. PFMT is performed under controlled and supervised conditions, or as a home training programme [10, 11], and can be performed with or without assistive devices such as electrical stimulation (ES) and biofeedback (BFB) [12]. Abdominal muscle recruitment, combined or not combined with PFMT, is also a common approach for UI because the coordinated and balanced activity of the abdominal muscles may act as a pressure mediator during activities that increase intra‐abdominal pressure, thus maintaining continence [13]. In addition, the deep abdominal and pelvic floor muscles are synergistic, so cocontractions of the abdominal muscles are usually observed when the pelvic floor is contracted [14].

Although recent guidelines recommend PFMT as a first‐line treatment for UI [9, 15, 16], the results for the treatment of postpartum UI are inconclusive and insufficiently quantified, with previous evidence reporting beneficial effects [2] whereas others have reported uncertain results [17]. In addition, the literature to date has focused on PFMT without including other training strategies, such as abdominal muscle training (AMT), ES, and BFB, sometimes together with educational advice and recommendations, which are commonly used in combination with PFMT [18, 19].

Therefore, this systematic review and meta‐analysis aimed to (i) assess the efficacy of abdominal and/or PFMT combined with other conservative measures in the treatment of UI during the postpartum period, and (ii) determine whether factors such as maternal age, type and time from delivery, parity, and duration or frequency of treatment have any influence on the severity of UI.

## 2 Methods

### 2.1 Protocol Registration and Reporting

This study was conducted according to the Preferred Reporting Items for Systematic Reviews and Meta‐Analyses (PRISMA) guidelines [20] (Table S1) and the recommendations of the Cochrane Collaboration Handbook [21]. The protocol was previously registered on PROSPERO (registration number: CRD42023489312).

### 2.2 Search Methodology

Systematic searches in the MEDLINE (via PubMed), Scopus, Cochrane Database of Systematic Reviews, Web of Science and Physiotherapy Evidence Database (PEDro) databases were conducted from inception to November 6th, 2024, to identify experimental studies that aimed to determine the effects of training interventions in the management of postpartum UI. References for included articles were also reviewed. All studies were loaded into Zotero desktop, and the duplicate merger tool was used to search for duplicates. Further details of the search strategy used for each database are available in Table S2.

### 2.3 Study Inclusion and Exclusion Criteria

Eligible articles were experimental studies (randomised controlled trials (RCTs) or non‐RCTs and single‐arm pre–post studies) that aimed to measure the effectiveness of different types of conservative therapies in the physiotherapy treatment of postpartum UI. We excluded studies that were not written in English or Spanish and were ineligible for publication types, review articles, editorials, commentaries, guidelines, and case reports.

After the selection of the included studies, the conservative physiotherapy treatments were categorised as PFMT, PFMT through ES, BFB or both, PFMT+education, PFMT+AMT, and AMT.

Exercises aimed at increasing muscle strength, power, and endurance through selective contractions of the pelvic floor muscles are considered PFMT [22]. This training can be carried out in a medical setting under supervision or control, or at home without supervision. The different postural exercises used to work the abdominal wall, such as Pilates and the abdominal hypopressive technique, are termed AMT. ES is used to induce contractions of the pelvic floor muscles, thereby strengthening the muscle fibres [23]. The BFB supported voluntary control of muscle contraction by providing information about the muscle activity being performed through graphic and/or auditory signals [24]. Education included instructions on how to contract the pelvic floor muscles correctly [25], as well as dietary and lifestyle recommendations [26].

The severity of UI was measured via patient‐reported questionnaires or pad tests. When the studies reported the outcome measures via more than one tool, the validated questionnaires were selected as the first option, as they are more commonly used in research settings.

The literature search was conducted independently by two reviewers, and disagreements were resolved by consensus or discussion with a third researcher.

### 2.4 Data Extraction

Two authors independently extracted the following information from the original reports: (i) first author name and publication year; (ii) study type; (iii) study population characteristics (country, sample size, maternal age, time since delivery, delivery type, and parity); (iv) intervention characteristics (intervention, duration of treatment, and number of sessions); and (v) UI outcomes. A third researcher independently assessed the accuracy of the extracted information.

### 2.5 Risk of Bias Assessment

In the RCTs, the intervention arms were taken to be treated as pre‐post intervention studies, but they were assessed for methodology as RCTs. The methodological quality of the RCTs was assessed via the Cochrane Collaboration tool for assessing the risk of bias (RoB2) [27]. This tool assesses the risk of bias according to five domains: the randomization process, deviations from the intended interventions, missing outcome data, measurement of the outcome, and selection of the reported result. Overall, a study was considered to have a “low risk of bias” if all domains were considered to have a “low risk,” to have “some concerns” if there was at least one domain rated as having “some concerns,” and to have a “high risk of bias” if there was at least one domain rated as having a “high risk” or several domains rated as having “some concerns” that could affect the validity of the results. The methodological quality of the nonrandomized studies was assessed via the risk of bias in nonrandomized studies of intervention (ROBINS‐I) [28]. This tool assesses the risk of bias according to six domains: bias due to confounding, bias in the selection of participants, bias in the classification of interventions, bias due to deviations from the intended interventions, bias due to missing data, and bias in the selection of the reported results. Each domain could be considered low, moderate, or serious, with a critical risk of bias or no information. Overall bias is considered “low risk of bias” if all domains are rated as having a low risk of bias; “moderate risk of bias” if all domains are rated as having a low or moderate risk of bias; “serious risk of bias” if there is at least one domain rated as having serious risk; “critical risk of bias” if there is at least one domain rated as having critical risk; and “no information” if there is no clear indication that the study is at a serious or critical risk of bias and if there is a lack of information about one or more key domains of bias. The risk of bias was assessed independently by two reviewers (concordance agreement by weighted kappa statistics 0.6), and inconsistencies were resolved by consensus or discussion with a third researcher.

### 2.6 Statistical Analysis

The effectiveness of the interventions was estimated using two approaches. First, we calculated the pre‐post of the intervention arms using the standardised mean differences (SMDs) with their 95% confidence interval (95% CI) by Cohen's *d* statistic [29]. Second, when the studies were RCTs we estimated the differences between the intervention and comparison groups using also SMDs by Cohen's *d*, considering values of 0.2, 0.5, 0.8, and greater than 1.0 of this statistic as weak, moderate, strong, and very strong effects, respectively. Finally, we used the Hartung‐Knapp‐Sidik‐Jonkman procedure [30] to calculate the pooled estimates of these statistics and their 95% CI. This analysis was repeated via DerSimonian and Laird's method [31] to facilitate comparability of the results of our meta‐analysis with those of previously published review articles. Where negative SMD values indicate an improvement in the UI of the training intervention comparison group. When the studies included two intervention groups, their data were analysed as independent samples.

We used the *I* ^2^ statistic to assess the level of heterogeneity of the results among the studies. The level of heterogeneity was categorised as follows: may not be important (0%–40%), may represent moderate heterogeneity (30%–60%), may indicate substantial heterogeneity (50%–90%), or may indicate considerable heterogeneity (75%–100%). The corresponding *p* values were also considered [32].

We conducted a sensitivity analysis by removing studies one by one to assess the robustness of the summary estimates.

Subgroup analyses were performed considering the Cohen's *d* of the intervention groups according to the following categorical variables: characteristics of the population (maternal age, time from delivery, delivery type, type of patients), characteristics of the intervention (duration of treatment and number of sessions), and type of measurement (objective or subjective). In addition, random‐effects meta‐regression models were conducted to assess the influence of the continuous variables that were potential confounders (maternal age, time after delivery, length of the interventions, and number of sessions). We assessed publication bias via Egger's regression asymmetry test [33], and *p* values < 0.10 were considered statistically significant. Statistical analyses were performed via R software V4.2.1.

## 3 Results

### 3.1 Systematic Review

**FIGURE 1:**
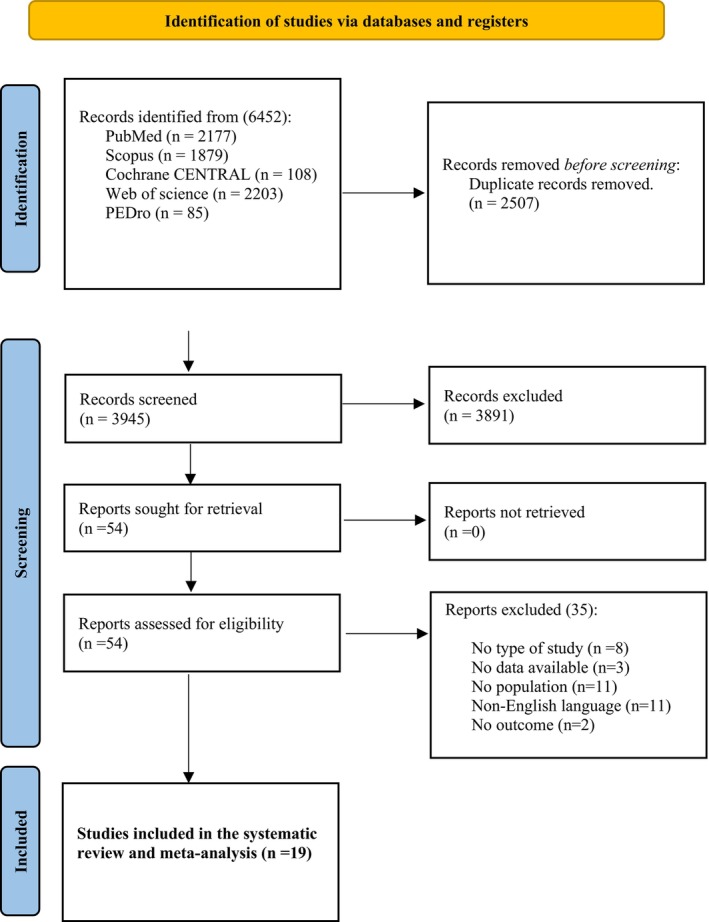
Flow diagram.

Nineteen studies were identified (16 RCTs and 3 pre‐post studies) (Figure 1), including 2737 participants (excluded studies and reasons are provided in Table S3). The studies were conducted on five continents: North America [34], South America [35], Europe [36, 37, 38, 39], Asia [40, 41, 42, 43, 44, 45, 46, 47, 48], Oceania [49, 50] and Africa [51, 52]. Participants' ages ranged from 25.0 to 50.37 years, with sample sizes ranging from 9 to 371 subjects (Table S4). The time elapsed from the time the participants gave birth until the intervention was initiated ranged from 0.25 to 48 weeks. A total of 26.31% of the included studies were conducted with only primiparous women, 5.26% only non‐primiparous women, 31.57% with both primiparous and non‐primiparous women, and 36.84% of the studies did not report this information. In addition, 47.36% of the women included in the studies had a vaginal delivery, 42.10% had a vaginal delivery or a caesarean section, and 10.52% of the studies did not report this.

The interventions included several types of training: eleven were PFMT [35, 37, 38, 40, 41, 43, 44, 49, 51], nine interventions were PFMT through ES/BFB [34, 39, 40, 43, 44, 46, 48], five interventions consisted of PFMT combined with AMT [34, 42, 51, 52], six interventions were described as PFMT combined with education [36, 45, 47, 48, 50], and two interventions were AMT [37, 45]. The length of the interventions ranged from 4 to 36 weeks, with a total number of sessions between < 24 and > 40.

With respect to the methodology used to assess UI, the studies used a range of instruments, including the International Consultation on Incontinence‐Short Form Questionnaire (ICIQ‐SF) [35, 37, 40, 41, 44, 45, 46, 48, 51, 52]; the Bristol Female Lower Urinary Tract Symptoms Questionnaire (BFLUTS) [36, 42]; a self‐reported satisfaction survey [38]; the Urinary Distress Inventory (UDI) [34]; the Visual Analogue Scale (VAS), [47]; and the Australian Pelvic Floor Questionnaire [39]; or objective measurements such as the volume total vesical (VTV), [43], and the pad test, [49].

### 3.2 Study Quality

The overall risk of bias for RCTs, as assessed by the RoB2 tool, showed some concerns for all included studies (mainly related to the selection of the reported results and the measurement of the outcome domains) (Figure S1). The overall risk of bias for the non‐RCTs, as assessed by the ROBINS‐I tool, showed a moderate risk of bias in 33.3% of the included studies and a serious risk of bias in 66.6% of the included studies (mainly due to a serious risk of bias due to confounding) (Figure S2).

### 3.3 Meta‐Analysis

**FIGURE 2:**
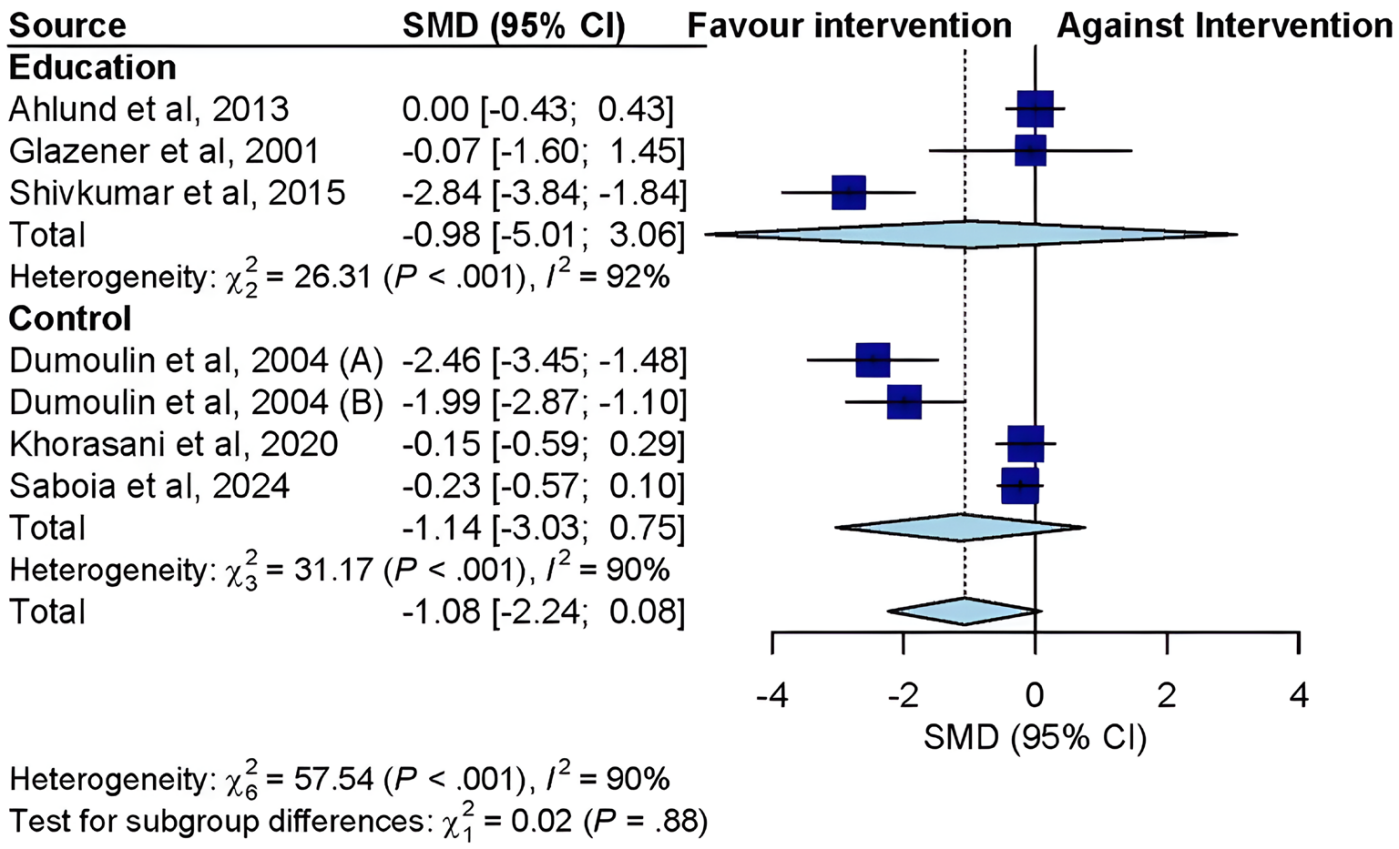
Forest plot of randomised controlled trials showing the effect size of training interventions on UI during the postpartum period versus education or control conditions.

The analysis of RCTs evaluating the effect of physiotherapy treatments through training interventions versus the comparison group (control group or educational intervention) revealed no statistically significant difference in the improvement of postpartum UI (Cohen's *d* = −1.08; 95% CI: −2.24 to 0.08; *I* ^2^ = 90%). For the analysis of the exercise intervention group versus the education group (Cohen's *d* = −0.98; 95% CI: −5.01 to 3.06) and the control group (Cohen's *d* = −1.14; 95% CI: −3.03 to 0.75), a nonsignificant reduction in the UI was observed, both with considerable heterogeneity (*I* ^2^ = 92%; *p* < 0.001 and *I* ^2^ = 90%; *p* < 0.001, respectively) (Figure 2).

**FIGURE 3:**
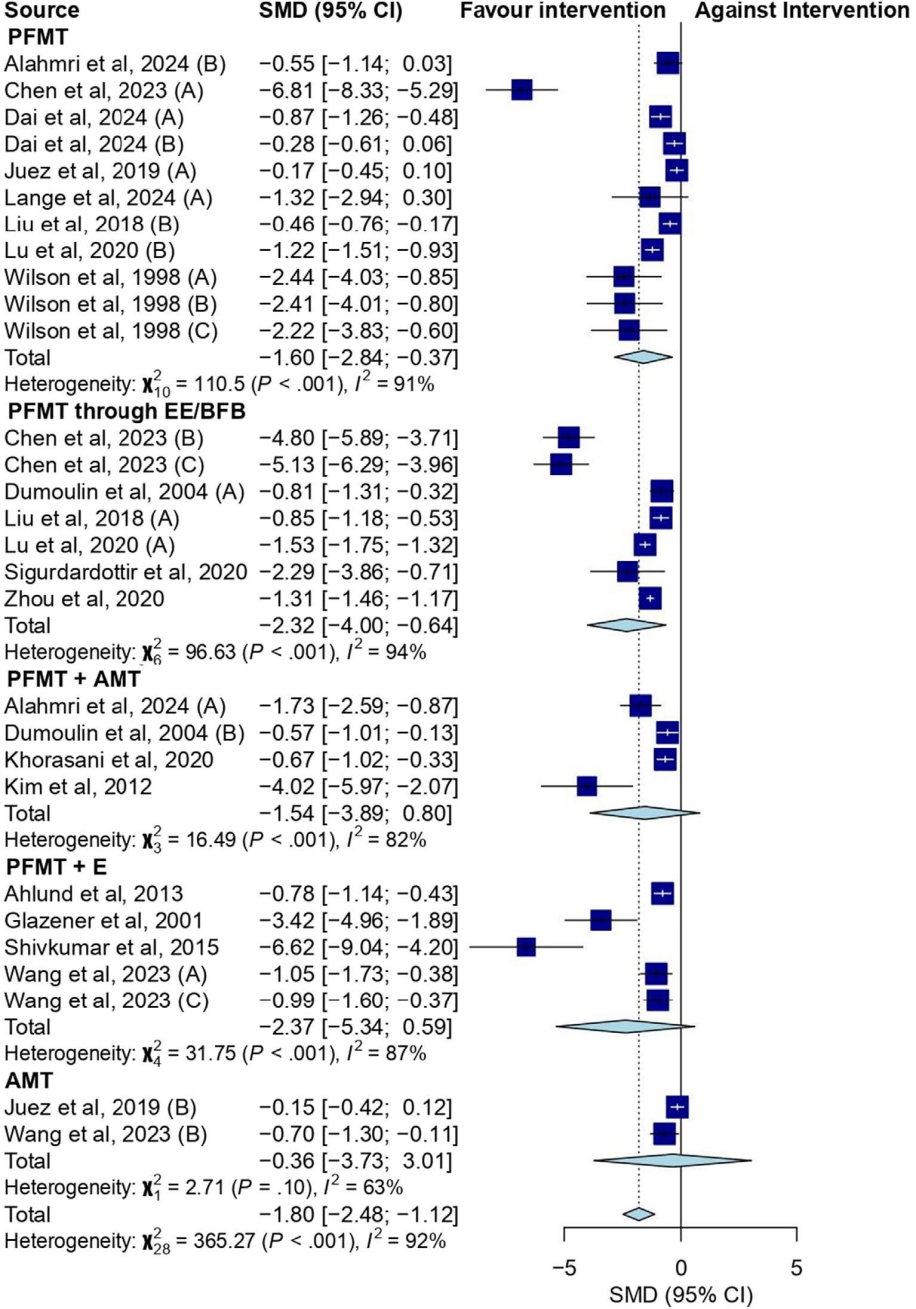
Forest plot showing the effect size of training interventions on UI during the postpartum period using.

When the Cohen's d was estimated considering only the effect on the intervention groups without a comparison group, there was a significant improvement in the UI (Cohen's *d* = −1.75; 95% CI: −2.37 to −1.13) with considerable heterogeneity (*I* ^2^ = 96%; *p* < 0.001). Furthermore, PFMT (Cohen's *d* = −1.45; 95% CI: −2.61 to −0.28), PFMT through EE/BFB (Cohen's *d* = −2.16; 95% CI: −3.50 to −0.81), and PFMT combined with AMT (Cohen's *d* = −1.73; 95% CI: −3.42 to −0.03) modalities showed a statistically significant reduction of UI in postpartum women, both with considerable heterogeneity (*I* ^2^ = 90%; *p* < 0.001, *I* ^2^ = 99%; *p* < 0.001, and *I* ^2^ = 83%; *p* < 0.001 respectively) (Figure 3).

#### 3.3.1 Subgroup Analyses Based on Population Characteristics

When maternal age was taken into account, the different training modalities were statistically significant reduction in UI in all age subgroups: less than 30 years (Cohen's *d* = −1.78; 95% CI: −2.90 to −0.66; *I* ^2^ = 97%), between 30 and 35 (Cohen's *d* = −2.20; 95% CI: −3.52 to −0.87; *I* ^2^ = 96%), and over 35 (Cohen's *d* = −1.03; 95% CI: −1.67 to −0.38; *I* ^2^ = 57%). A subgroup analysis based on time since delivery showed a statistically significant improvement in UI in mothers between six and 12 weeks postpartum (Cohen's *d* = −1.89; 95% CI: −3.09 to −0.68; *I* ^2^ = 98%) and more than 12 weeks postpartum (Cohen's *d* = −1.79; 95% CI: −2.76 to −0.82; *I* ^2^ = 76%). When the delivery type was controlled, the different training modalities showed significant improvements, regardless of type of delivery, in mothers who delivered vaginally (Cohen's *d* = −2.08; 95% CI: −3.25 to −0.92; *I* ^2^ = 98%) and who delivered vaginally or not (Cohen's *d* = −1.23; 95% CI: −1.71 to −0.75; *I* ^2^ = 91%). Finally, considering the parity, training modalities reduce the UI in primiparous (Cohen's *d* = −2.48; 95% CI: −4.70 to −0.27; *I* ^2^ = 97%) and in studies conducted in primiparous and multiparous (Cohen's *d* = −1.31; 95% CI: −1.89 to −0.73; *I* ^2^ = 93%) (Table S5a).

#### 3.3.2 Subgroup Analyses Based on the Intervention Characteristics

With respect to the duration of the intervention, significant improvements were found in treatments up to 8 weeks (Cohen's *d* = −1.53; 95% CI: −2.21 to −0.84; *I* ^2^ = 96%), as well as in treatments of 12 weeks (Cohen's *d* = −2.18; 95% CI: −3.70 to −0.65; *I* ^2^ = 97%) that showed a statistical reduction of the UI. In addition, interventions with less than 24 sessions (Cohen's *d* = −1.68; 95% CI: −2.43 to −0.93; *I* ^2^ = 96%), between 24 and 40 sessions (Cohen's *d* = −1.33; 95% CI: −2.41 to −0.25; *I* ^2^ = 95%), and interventions with more than 40 sessions (Cohen's *d* = −2.43; 95% CI: −4.37 to −0.50; *I* ^2^ = 94%) were shown to be effective in reducing UI (Table S5b).

#### 3.3.3 Subgroup Analyses by Type of Outcome Measure

In the subgroup analysis considering the method of measurement of the variable, statistically significant differences were found for both objective (Cohen's *d* = −1.74; 95% CI: −2.95 to −0.54; *I* ^2^ = 82%) and subjective (Cohen's *d* = −1.74; 95% CI: −2.48 to −0.99; *I* ^2^ = 97%) methods of measurement (Table S5c).

#### 3.3.4 Meta‐Regression Analyses

The random‐effects meta‐regression models showed only significant differences in the number of sessions of the intervention (*p* = 0.016) (Table S6).

### 3.4 Sensitivity Analysis

When the impact of the individual studies was examined by removing studies from the analysis one at a time (leave‐one‐out procedure), the pooled Cohen's d estimates for exercise interventions on UI did not change significantly (Figure S3). Furthermore, when analyses were repeated via DerSimonian and Laird's method, the overall results were similar to those found obtained via the Hartung‐Knapp‐Sidik‐Jonkman method (Cohen's *d* = −1.56; 95% CI: −1.91 to −1.22) with considerable heterogeneity (*I* ^2^ = 87%; *p* < 0.001). In addition, all training interventions except AMT isolated showed a significant improvement in postpartum UI severity (Figure S4). The analyses of RCTs with this method also showed a significant difference in favour of the intervention (Cohen's *d* = −1.05; 95% CI: −1.77 to −0.32) with considerable heterogeneity (*I* ^2^ = 90%; *p* < 0.001) (Figure S5).

### 3.5 Publication Bias

The asymmetry of the funnel plot and Egger's test suggested that there was publication bias in the pre and postintervention analyses for the UI (*p* = 0.045) (Figure S6).

## 4 Discussion

### 4.1 Main Findings

Our data supports that training interventions, mainly PFMT, combined with ES, BFB, or AMT, are effective in the physiotherapy treatment of postpartum UI. Potential effect modifiers related to the characteristics of the population, the intervention, or the outcome measurement tool outcome did not change our estimates, although it seems that intensive treatments (more sessions) over 12 weeks had a greater beneficial effect on reducing UI symptoms.

### 4.2 Interpretation

Current evidence suggests that PFMT improves strength, muscle building, and pelvic floor structural support and helps to restore intra‐abdominal pressure to normal [53]. The literature has focused on the prevention of UI both during pregnancy and in the postpartum period with positive results [54, 55]. Our data also support these positive effects for the treatment of postpartum UI via this type of training in isolation or with other tools such as ES or BFB. The rationale for this may be that ES can induce muscle contractions that hypertrophy or increase the number of muscle fibres and contribute to the reorganisation of collagen in the pelvic floor musculature [56], and BFB makes women aware of the work they are doing, resulting in increased sensitivity, strength, and coordination [57]. Notably, no adverse effects have been reported with this type of training [38, 39].

The synergy between the pelvic floor musculature and the abdominal wall is well known [58, 59], and training these structures is important for improving the stabilisation of the trunk and the lumbo‐pelvic area, as well as increasing the activation of the pelvic floor muscles, which contributes to the maintenance of urinary continence [60]. Coordinated work can thus be achieved to prevent the urethra from descending during increases in intra‐abdominal pressure [16]. Our data support these findings, and PFMT combined with AMT showed a significant positive impact on UI. However, our pooled assessment of the effect of AMT in isolation showed a positive trend but not significant differences, probably because of the limited number of included studies.

The combination of PFMT with other interventions such as education, although it tends to favour effects, did not significantly differ, which may be due to the small sample sizes [36, 61] and the heterogeneity of the interventions in the included studies. In this regard, it is noteworthy that these differences were observed when the analyses were repeated via the DerSimonian and Laird method for all training interventions except AMT; thus, that further studies may help to identify significant differences.

### 4.3 Strengths and Limitations

Some of the studies included in this review had large effect sizes [40, 42, 47, 50] probably because of the small sample size [40, 42, 47] but also because of other methodological characteristics such as the intensity of the treatment, which was 8–10 sessions per day [50], which may have influenced our results.

Our findings showed positive results for physiotherapy treatments through training interventions for postpartum UI management, but no differences were observed regarding population characteristics (maternal age, time from delivery, type, and number of deliveries) or the way the outcome was measured (objective and subjective). In addition, regardless of the duration of the training, there were also positive effects on the severity of UI, although more intensive and longer treatments seemed to have a greater effect on symptomatology.

This systematic review and meta‐analysis has several limitations that must be acknowledged to fully appreciate the extent of the estimates presented. The primary limitation is related to the scarcity of RCTs, the disparity of their designs, and their small sample sizes. However, based on the existing research, it would be redundant to conduct a meta‐analysis on the isolated effect of PFMT to prevent UI during pregnancy or the postpartum period, without making an effort to present comparisons between the different types of training interventions, combined or not with PFMT, which are commonly used in standard practice to treat UI after childbirth. For this reason, we comprehensively synthesised the existing data into a meta‐analysis to present pooled estimates. Second, there were large heterogeneities in the characteristics of the population, the characteristics of the interventions, or the way in which the severity of UI was measured. We conducted subgroup analyses, and the effect was only slightly modified; however, more homogeneous studies would allow more robust conclusions to be drawn. Finally, psychological determinants have emerged as relevant factors associated with UI in postpartum women, so these factors should be considered in the analyses of future studies [62].

## 5 Conclusions

Our meta‐analysis allows us to conclude that physiotherapy treatments through training interventions such as PFMT alone or combined with ES, BFB or AMT appear to be effective and clinically relevant and should be considered a first‐line treatment option for postpartum UI. However, further research is needed to achieve studies with more homogeneous designs and using standardised measurement methods, especially RCTs that investigate the importance of postural exercises that work on the abdominal and pelvic floor muscle synergy with the aim of improving continence.

## Author Contributions

**Cristina Gallego‐Gómez, Sergio de Núñez de Arenas‐Arroyo, Ana Torres‐Costoso, Asunción Ferri‐Morales** and **Vicente Martínez‐Vizcaíno:** conception and design of the work. **Cristina Gallego‐Gómez:** data curation, writing – original draft preparation. **Sergio de Núñez de Arenas‐Arroyo:** conceptualisation, methodology, software. **Ana Torres‐Costoso:** visualisation, investigation. **Vicente Martínez‐Vizcaíno:** supervision. **Sandra Martínez‐Bustelo, Claudia Andrea Quezada‐Bascuñán, Julián Ángel Basco‐López** and **Eva Rodríguez‐Gutiérrez:** software, validation. **Asunción Ferri‐Morales:** writing – reviewing and editing. All authors made significant contributions to drafting and/or revising the article. All authors approved the final version of the article for publication.

## Ethics Statement

The authors have nothing to report.

## Conflicts of Interest

The authors declare no conflicts of interest.

## Supporting information


**Data S1:** bjo70014‐sup‐0001‐DataS1.docx.

## Data Availability

The data that support the findings of this study are available in the Supporting Information for this article.
