# Supplementary material for: Effects of Training Interventions to Treat Postpartum Urinary Incontinence: A Meta‐Analysis
Source: BJOG. 2025 Sep 24;133(2):243–52. doi: 10.1111/1471-0528.70014 (PMC12678044; doi:10.1111/1471-0528.70014)

**Effects of training interventions to treat postpartum urinary incontinence: a meta-analysis**

**Table S1.** Preferred Reporting Items for Systematic Reviews and Meta-Analyses (PRISMA Guide).

**Table S2.** Database search strategy.

**Table S3.** Excluded studies with reasons for exclusion.

**Table S4.** Characteristics of the included studies.

**Table S5.** Subgroup analyses by:

1. Characteristics of population (**Table S5a**)
2. Characteristics of the intervention (**Table S5b**)
3. Type of outcome measure (**Table S5c**)

**Table S6.** Random effects meta-regression models

**Figure S1.** Risk of bias of randomized controlled trials studies (ROB-2)

**Figure S2**. Risk of bias of non-randomized studies (ROBINS-I)

**Figure S3.** Sensitivity analysis.

**Figure S4**. Forest plot showing the effect size of pre-post training interventions on UI during postpartum period using Der Simonian and Laird method.

**Figure S5.** Forest plot of randomized controlled trials showing the effect size of training interventions on UI during postpartum period versus education or control conditions using Der Simonian and Laird method.

**Figure S6.** Funnel plot.

**Table S1.** Preferred Reporting Items for Systematic Reviews and Meta-Analyses (PRISMA Guide).

PRISMA 2020 checklist.

|  |  | Reporting Item | Page Number |
| --- | --- | --- | --- |
| **Title** |  |  |  |
| Title | [#1](https://www.goodreports.org/reporting-checklists/prisma/info/#1) | Identify the report as a systematic review | 1 |
| **Abstract** |  |  |  |
| Abstract | [#2](https://www.goodreports.org/reporting-checklists/prisma/info/#2) | Report an abstract addressing each item in the PRISMA 2020 for Abstracts checklist | 3 and 4 |
| **Introduction** |  |  |  |
| Background/rationale | [#3](https://www.goodreports.org/reporting-checklists/prisma/info/#3) | Describe the rationale for the review in the context of existing knowledge | 5 and 6 |
| Objectives | [#4](https://www.goodreports.org/reporting-checklists/prisma/info/#4) | Provide an explicit statement of the objective(s) or question(s) the review addresses | 6 |
| **Methods** |  |  |  |
| Eligibility criteria | [#5](https://www.goodreports.org/reporting-checklists/prisma/info/#5) | Specify the inclusion and exclusion criteria for the review and how studies were grouped for the syntheses | 7 |
| Information sources | [#6](https://www.goodreports.org/reporting-checklists/prisma/info/#6) | Specify all databases, registers, websites, organisations, reference lists, and other sources searched or consulted to identify studies. Specify the date when each source was last searched or consulted | 6 |
| Search strategy | [#7](https://www.goodreports.org/reporting-checklists/prisma/info/#7) | Present the full search strategies for all databases, registers, and websites, including any filters and limits used | Table S2 |
| Selection process | [#8](https://www.goodreports.org/reporting-checklists/prisma/info/#8) | Specify the methods used to decide whether a study met the inclusion criteria of the review, including how many reviewers screened each record and each report retrieved, whether they worked independently, and, if applicable, details of automation tools used in the process | 8 |
| Data collection process | [#9](https://www.goodreports.org/reporting-checklists/prisma/info/#9) | Specify the methods used to collect data from reports, including how many reviewers collected data from each report, whether they worked independently, any processes for obtaining or confirming data from study investigators, and, if applicable, details of automation tools used in the process | 8 |
| Data items | [#10a](https://www.goodreports.org/reporting-checklists/prisma/info/#10a) | List and define all outcomes for which data were sought. Specify whether all results that were compatible with each outcome domain in each study were sought (for example, for all measures, time points, analyses), and, if not, the methods used to decide which results to collect | 9 and 10 |
| Study risk of bias assessment | [#11](https://www.goodreports.org/reporting-checklists/prisma/info/#11) | Specify the methods used to assess risk of bias in the included studies, including details of the tool(s) used, how many reviewers assessed each study and whether they worked independently, and, if applicable, details of automation tools used in the process | 8 and 9 |
| Effect measures | [#12](https://www.goodreports.org/reporting-checklists/prisma/info/#12) | Specify for each outcome the effect measure(s) (such as risk ratio, mean difference) used in the synthesis or presentation of results | 9 and 10 |
| Synthesis methods | [#13a](https://www.goodreports.org/reporting-checklists/prisma/info/#13a) | Describe the processes used to decide which studies were eligible for each synthesis (such as tabulating the study intervention characteristics and comparing against the planned groups for each synthesis (item #5)) | 7 and 8 |
| Synthesis methods | [#13b](https://www.goodreports.org/reporting-checklists/prisma/info/#13b) | Describe any methods required to prepare the data for presentation or synthesis, such as handling of missing summary statistics or data conversions | 7 and 8 |
| Synthesis methods | [#13c](https://www.goodreports.org/reporting-checklists/prisma/info/#13c) | Describe any methods used to tabulate or visually display results of individual studies and syntheses | 7 and 8 |
| Synthesis methods | [#13d](https://www.goodreports.org/reporting-checklists/prisma/info/#13d) | Describe any methods used to synthesise results and provide a rationale for the choice(s). If meta-analysis was performed, describe the model(s), method(s) to identify the presence and extent of statistical heterogeneity, and software package(s) used | 9 and 10 |
| Synthesis methods | [#13e](https://www.goodreports.org/reporting-checklists/prisma/info/#13e) | Describe any methods used to explore possible causes of heterogeneity among study results (such as subgroup analysis, meta-regression) | 10 |
| Synthesis methods | [#13f](https://www.goodreports.org/reporting-checklists/prisma/info/#13f) | Describe any sensitivity analyses conducted to assess robustness of the synthesised results | 10 |
| Reporting bias assessment | [#14](https://www.goodreports.org/reporting-checklists/prisma/info/#14) | Describe any methods used to assess risk of bias due to missing results in a synthesis (arising from reporting biases) | 8 and 9 |
| Certainty assessment | [#15](https://www.goodreports.org/reporting-checklists/prisma/info/#15) | Describe any methods used to assess certainty (or confidence) in the body of evidence for an outcome | NR |
| Data items | [#10b](https://www.goodreports.org/reporting-checklists/prisma/info/#10b) | List and define all other variables for which data were sought (such as participant and intervention characteristics, funding sources). Describe any assumptions made about any missing or unclear information | 8 |
| **Results** |  |  |  |
| Study selection | [#16a](https://www.goodreports.org/reporting-checklists/prisma/info/#16a) | Describe the results of the search and selection process, from the number of records identified in the search to the number of studies included in the review, ideally using a flow diagram (http://www.prisma-statement.org/PRISMAStatement/FlowDiagram) | 10 and 11 Figure 1 |
| Study selection | [#16b](https://www.goodreports.org/reporting-checklists/prisma/info/#16b) | Cite studies that might appear to meet the inclusion criteria, but which were excluded, and explain why they were excluded | Table S3 |
| Study characteristics | [#17](https://www.goodreports.org/reporting-checklists/prisma/info/#17) | Cite each included study and present its characteristics | 10 and 11 Table 1 |
| Risk of bias in studies | [#18](https://www.goodreports.org/reporting-checklists/prisma/info/#18) | Present assessments of risk of bias for each included study | 12 and Table S5 and S6 |
| Results of individual studies | [#19](https://www.goodreports.org/reporting-checklists/prisma/info/#19) | For all outcomes, present for each study (a) summary statistics for each group (where appropriate) and (b) an effect estimate and its precision (such as confidence/credible interval), ideally using structured tables or plots | Fig. 2 and Table S4, S8 and S9 |
| Results of syntheses | [#20a](https://www.goodreports.org/reporting-checklists/prisma/info/#20a) | For each synthesis, briefly summarise the characteristics and risk of bias among contributing studies | 12 and Table S5 and S6 |
| Results of syntheses | [#20b](https://www.goodreports.org/reporting-checklists/prisma/info/#20b) | Present results of all statistical syntheses conducted. If meta-analysis was done, present for each the summary estimate and its precision (such as confidence/credible interval) and measures of statistical heterogeneity. If comparing groups, describe the direction of the effect | 12, 13 and 14. Fig. 2, Table 2 and Table S4, S8 and S9 |
| Results of syntheses | [#20c](https://www.goodreports.org/reporting-checklists/prisma/info/#20c) | Present results of all investigations of possible causes of heterogeneity among study results | NR |
| Results of syntheses | [#20d](https://www.goodreports.org/reporting-checklists/prisma/info/#20d) | Present results of all sensitivity analyses conducted to assess the robustness of the synthesised results | 14 and Table S7 |
| Risk of reporting biases in syntheses | [#21](https://www.goodreports.org/reporting-checklists/prisma/info/#21) | Present assessments of risk of bias due to missing results (arising from reporting biases) for each synthesis assessed | 14 and Table S10 |
| Certainty of evidence | [#22](https://www.goodreports.org/reporting-checklists/prisma/info/#22) | Present assessments of certainty (or confidence) in the body of evidence for each outcome assessed | NR |
| **Discussion** |  |  |  |
| Results in context | [#23a](https://www.goodreports.org/reporting-checklists/prisma/info/#23a) | Provide a general interpretation of the results in the context of other evidence | 14, 15 and 16 |
| Limitations of included studies | [#23b](https://www.goodreports.org/reporting-checklists/prisma/info/#23b) | Discuss any limitations of the evidence included in the review | 16 and 17 |
| Limitations of the review methods | [#23c](https://www.goodreports.org/reporting-checklists/prisma/info/#23c) | Discuss any limitations of the review processes used | 16 and 17 |
| Implications | [#23d](https://www.goodreports.org/reporting-checklists/prisma/info/#23d) | Discuss implications of the results for practice, policy, and future research | 14, 15 and 16 |
| **Other information** |  |  |  |
| Registration and protocol | [#24a](https://www.goodreports.org/reporting-checklists/prisma/info/#24a) | Provide registration information for the review, including register name and registration number, or state that the review was not registered | 2 and 6 |
| Registration and protocol | [#24b](https://www.goodreports.org/reporting-checklists/prisma/info/#24b) | Indicate where the review protocol can be accessed, or state that a protocol was not prepared | NR |
| Registration and protocol | [#24c](https://www.goodreports.org/reporting-checklists/prisma/info/#24c) | Describe and explain any amendments to information provided at registration or in the protocol | NR |
| Support | [#25](https://www.goodreports.org/reporting-checklists/prisma/info/#25) | Describe sources of financial or non-financial support for the review, and the role of the funders or sponsors in the review | 1 |
| Competing interests | [#26](https://www.goodreports.org/reporting-checklists/prisma/info/#26) | Declare any competing interests of review authors | 1 |
| Availability of data, code, and other materials | [#27](https://www.goodreports.org/reporting-checklists/prisma/info/#27) | Report which of the following are publicly available and where they can be found: template data collection forms; data extracted from included studies; data used for all analyses; analytic code; any other materials used in the review | NR |

**Table S2.** Database search strategy.

| **Search set for Medline (via Pudmed)** |
| --- |
| ("urinary Incontinence" OR "Urinary disorders" OR "Reflex Urinary Incontinence" OR "Urge Urinary Incontinence" OR "Stress Urinary Incontinence" OR "Mixed Urinary Incontinence" OR "urine loss" OR "leaking urine" OR "incontinence" OR "lower urinary tract symptoms" OR "Pelvic Floor Disorders" OR "Urinary Incontinence"[Mesh] OR "Urinary Incontinence, Urge"[Mesh] OR "Urinary Incontinence, Stress"[Mesh]OR "Lower Urinary Tract Symptoms"[Mesh]OR "Pelvic Floor Disorders"[Mesh]) AND ("after birth" OR "after delivery" OR "post natal" OR postnatal OR puerperal OR childbirth OR "after childbirth" OR postpartum OR "postpartum period" OR postdelivery OR postlabor) AND (treatment OR therapy OR treat* OR procedure OR exercise OR "pelvic floor muscle exercise" OR "pelvic floor muscle training" OR "pelvic diaphragm*" OR Kegel OR biofeedback OR "resistance training" OR " feedback" OR "electrical stimulat*" OR electrostimulat* OR "electric stimulation therapy" OR "pelvic floor rehabilitation" OR kinesiotherap* OR "physical therap*" OR physiotherap* OR "conservative treatment" OR rehabilitation OR "myofunctional therapy" OR "exercise therapy" OR "muscle training" OR "Physical Therapy Modalities"[Mesh] OR "Rehabilitation"[Mesh]) |
| **Total results**: 2177 |
| **Search set for Scopus** |
| ("urinary Incontinence" OR "Urinary disorders" OR "Reflex Urinary Incontinence" OR "Urge Urinary Incontinence" OR "Stress Urinary Incontinence" OR "Mixed Urinary Incontinence" OR "urine loss" OR "leaking urine" OR "incontinence" OR "lower urinary tract symptoms" OR "Pelvic Floor Disorders") AND ("after birth" OR "after delivery" OR postnatal OR puerperal OR childbirth OR "after childbirth" OR postpartum OR postdelivery OR postlabor) AND (treatment OR therapy OR procedure OR exercise OR "pelvic floor muscle exercise" OR "pelvic floor muscle training" OR Kegel OR biofeedback OR "resistance training" OR "electrical stimulat*" OR electrostimulat* OR "pelvic floor rehabilitation" OR kinesiotherap* OR "physical therap*" OR physiotherap* OR "conservative treatment" OR rehabilitation OR "myofunctional therapy" OR "exercise therapy" OR "muscle training" OR "Physical Therapy Modalities" OR "Rehabilitation") |
| **Total results**: 1879 |
| **Search set for Cochrane CENTRAL** |
| ("urinary Incontinence" OR "Urinary disorders" OR "Reflex Urinary Incontinence" OR "Urge Urinary Incontinence" OR "Stress Urinary Incontinence" OR "Mixed Urinary Incontinence" OR "urine loss" OR "leaking urine" OR "incontinence" OR "lower urinary tract symptoms" OR "Pelvic Floor Disorders") AND ("after birth" OR "after delivery" OR postnatal OR puerperal OR childbirth OR "after childbirth" OR postpartum OR postdelivery OR postlabor) AND (treatment OR therapy OR procedure OR exercise OR "pelvic floor muscle exercise" OR "pelvic floor muscle training" OR Kegel OR biofeedback OR "resistance training" OR "electrical stimulat*" OR electrostimulat* OR "pelvic floor rehabilitation" OR kinesiotherap* OR "physical therap*" OR physiotherap* OR "conservative treatment" OR rehabilitation OR "myofunctional therapy" OR "exercise therapy" OR "muscle training" OR "Physical Therapy Modalities" OR "Rehabilitation") |
| **Total results**: 108 |
| **Search set for Web of Science** |
| ("urinary Incontinence" OR "Urinary disorders" OR "Reflex Urinary Incontinence" OR "Urge Urinary Incontinence" OR "Stress Urinary Incontinence" OR "Mixed Urinary Incontinence" OR "urine loss" OR "leaking urine" OR "incontinence" OR "lower urinary tract symptoms" OR "Pelvic Floor Disorders") AND ("after birth" OR "after delivery" OR postnatal OR puerperal OR childbirth OR "after childbirth" OR postpartum OR postdelivery OR postlabor) AND (treatment OR therapy OR procedure OR exercise OR "pelvic floor muscle exercise" OR "pelvic floor muscle training" OR Kegel OR biofeedback OR "resistance training" OR "electrical stimulat*" OR electrostimulat* OR "pelvic floor rehabilitation" OR kinesiotherap* OR "physical therap*" OR physiotherap* OR "conservative treatment" OR rehabilitation OR "myofunctional therapy" OR "exercise therapy" OR "muscle training" OR "Physical Therapy Modalities" OR "Rehabilitation") |
| **Total results**: 2203 |
| **Search set for PEDro** |
| "incontinence" AND "postpartum" |
| **Total results**: 85 |

**Table S3.** Excluded studies with reasons for exclusion.

| **Study** | **Reason for exclusion** |
| --- | --- |
| Arshad S, Waseem I, Mahmood T, Batool F. Effects of Physical therapy Management of Stress Urinary Incontinence among Postnatal Women-A quasi experimental study. J Univ Med Dent Coll. 2022;13(2):377-81. | No data available |
| Artymuk NV; Khapacheva SY. Device-assisted pelvic floor muscle postpartum exercise programme for the management of pelvic floor dysfunction after delivery. The Journal of Maternal-Fetal & Neonatal Medicine 2022;35(3):481-485. 2022; | Population |
| Aston B. Postnatal pelvic floor dysfunction: conservative treatment and management options. J Fam Health Care. 2010;20(3):90-2. | No type of study |
| Chen Y, Pei H, Chen B. Evaluation of the effect of postpartum electrical stimulation and biofeedback combined with vaginal dumbbell exercise in improving pelvic floor function. Chin J Rehabil Med. 2013;28(3):234-237+257. | Non-English language |
| Chen F, Zhou J, Wu W, Qian X. Study on the therapeutic effect of floating needle therapy combined with pressing acupoint embedding for female stress urinary incontinence after childbirth: a randomized trial. Ann Palliat Med. julio de 2021;10(7):7786-93. | No type of study |
| 3A physiotherapist-led programme of postpartum pelvic floor exercises reduced urinary incontinence at 3 months. Evid-based Obstet Gynecol. 2003;5(1):38-9. | Population |
| Chu L, Jin X, Wu S, Tong X, Li H, Chen X. Effect of Pelvic Floor Muscle Training With Smartphone Reminders on Women in the Postpartum Period: A Randomized Controlled Trial. Urogynecology (Phila). 1 de febrero de 2024;30(2):138-46. | Population |
| Dumoulin C, Bourbonnais D, Morin M, Gravel D, Lemieux MC. Predictors of success for physiotherapy treatment in women with persistent postpartum stress urinary incontinence. Arch Phys Med Rehabil. julio de 2010;91(7):1059-63. | No type of study |
| Ewings P, Spencer S, Marsh H, O’Sullivan M. Obstetric risk factors for urinary incontinence and preventative pelvic floor exercises: cohort study and nested randomized controlled trial. J Obstet Gynaecol. agosto de 2005;25(6):558-64. | Education |
| Hagovská M, Urdzík P, Svihra J. Possibilities of objectivization of pelvic floor muscle exercises in patients with urine leakage after delivery. Ceska Gynekol. Winter de 2020;85(2):94-102. | Non-English language |
| Hilde G, Stær-Jensen J, Siafarikas F, Ellström Engh M, Bø K. Postpartum pelvic floor muscle training and urinary incontinence: a randomized controlled trial. Obstet Gynecol. diciembre de 2013;122(6):1231-8. | Population |
| KAORI Kinouchi. Pelvic Floor Muscle Training (PFMT) Reminder E-mail System using cellular phone to treat and prevent urinary incontinence in postnatal women. 2010; | No type of study |
| Lee IS, Choi ES. Pelvic floor muscle exercise by biofeedback and electrical stimulation to reinforce the pelvic floor muscle after normal delivery. Taehan Kanho Hakhoe Chi. diciembre de 2006;36(8):1374-80. | Population |
| Li F, Wang K, Liu K, Ebrahimi M. The Application of Pelvic Floor Ultrasound Image Analysis Technology in the Neurorehabilitation of Postpartum Urinary Incontinence. JOURNAL OF MEDICAL IMAGING AND HEALTH INFORMATICS. febrero de 2021;11(2):618-22. | Non-English language |
| Li Y, Bai W, Zhang J, Zhang R. Biofeedback Electrical Stimulation Therapy in the Rehabilitation of Pelvic Floor Function after Vaginal Delivery：a Clinical Randomized Controlled Study. Chin Gen Pract. 2024;27(5):547-51. | Non-English language |
| Liu DC, Gao L, Liu Y, Wang J, Li Y, Xu RY. Effect of different interventions on the efficacy of postpartum urinary incontinence in China: A systematic review and network meta-analysis. Medicine (Baltimore). 6 de octubre de 2023;102(40):e35473. | Non-English language |
| Monteiro MN, Micussi MTABC, Cruz VT da, Oliveira MC de, Medeiros KS, Sarmento ACA, et al. Pelvic floor muscle training program for women in the puerperal period: clinical progress after intervention. Rev Assoc Med Bras (1992). junio de 2021;67(6):851-6. | No type of study |
| Movahedi M; Torabipoor MS; Mohammadi MS; Shariat M; Haghollahi F; Hajihashem M. (The effect of postpartum physiotherapy on sexual function and incontinence of primparous women in al-Zahra and Shahid Beheshti Hospitals of Isfahan: a randomized clinical trial) [Persian]. Tehran University Medical Journal 2021 Aug;79(5):351-360. 2021; | Non-English language |
| Nahid R, Mahsa B, Alimohammadi N, Samar M. The Effect of Kegel Exercises and Pelvic Floor Physiotherapy on the Improvements of Stress Urinary Incontinence and Urge Incontinence in Women with Normal Vaginal Delivery. *Current Women’s Health Reviews*. 2024;20(4):1-5. | No data available |
| Alonso Perez L. Effectiveness of a pelvic floor muscle exercise program in urinary incontinence after childbirth. ENFERMERIA CLINICA. septiembre de 2007;17(5):281-2. | No type of study |
| Rao L, Zhang L, Yuan J, Lu B. Effect of postpartum pelvic floor muscle training on improving pelvic floor function. J Shanghai Jiaotong Univ Med Sci. 2023;43(3):308-13. | Non-English language |
| Sacomori C; Zomkowski K; dos Passos Porto I; Cardoso FL; Sperandio FF. Adherence and effectiveness of a single instruction of pelvic floor exercises: a randomized clinical trial. International Urogynecology Journal 2020 May;31(5):951-959. 2020; | No data available |
| Sampselle CM, Miller JM, Mims BL, Delancey JO, Ashton-Miller JA, Antonakos CL. Effect of pelvic muscle exercise on transient incontinence during pregnancy and after birth. Obstet Gynecol. marzo de 1998;91(3):406-12. | Population |
| Sleep J, Grant A. Pelvic floor exercises in postnatal care. Midwifery. diciembre de 1987;3(4):158-64. | Population |
| Śnieżek A, Czechowska D, Curyło M, Głodzik J, Szymanowski P, Rojek A, et al. Physiotherapy according to the BeBo Concept as prophylaxis and treatment of urinary incontinence in women after natural childbirth. Sci Rep. 10 de septiembre de 2021;11(1):18096. | Population |
| Tennfjord MK, Engh ME, Bø K. The Influence of Early Exercise Postpartum on Pelvic Floor Muscle Function and Prevalence of Pelvic Floor Dysfunction 12 Months Postpartum. Phys Ther. 31 de agosto de 2020;100(9):1681-9. | No outcome |
| Toal C, Goodman N, Durst R, Giugale L. Pelvic Floor Physical Therapy Attendance Among High-Risk Postpartum Patients. *Urogynecology (Phila)*. 2024;30(3):363-368. doi:10.1097/SPV.0000000000001492 | No outcome |
| Vesting S, Gutke A, Fagevik Olsén M, Rembeck G, Larsson MEH. The Impact of Exercising on Pelvic Symptom Severity, Pelvic Floor Muscle Strength, and Diastasis Recti Abdominis After Pregnancy: A Longitudinal Prospective Cohort Study. *Phys Ther*. 2024;104(4):pzad171. doi:10.1093/ptj/pzad171 | No type of study |
| Wang L-L; Ren Z-X; Zhu J-Y; Zhang H-L; Wu Y-R. (Efficacy of electroacupuncture combined with penetrating moxibustion for postpartum stress urinary incontinence) [Chinese - simplified characters]. Zhongguo Zhen Jiu [Chinese Acupuncture & Moxibustion] 2019 Jun;39(6):599-603. 2019; | Non-English language |
| Wang X, Xu X, Luo J, Chen Z, Feng S. Effect of app-based audio guidance pelvic floor muscle training on treatment of stress urinary incontinence in primiparas: A randomized controlled trial. Int J Nurs Stud. abril de 2020;104:103527. | Population |
| Wen X-H; Shi S-Q; Wang J-Y. (Pelvic muscles exercise for postpartum stress urinary incontinence) [Chinese - simplified characters]. Zhongguo Shiyong Yiyao [China Practical Medicine] 2010 May;5(15):72-73. 2010; | Non-English language |
| Yang YP, Liu YL, Shi YY, Liu XY. Effects of Pelvic Floor Muscle Training and Training Opportunity on Postpartum Pelvic Floor Function. Chin Gen Pract. 2016;19(26):3170-4. | Non-English language |
| Yang S, Sang W, Feng J, Zhao H, Li X, Li P, et al. The effect of rehabilitation exercises combined with direct vagina low voltage low frequency electric stimulation on pelvic nerve electrophysiology and tissue function in primiparous women: A randomised controlled trial. J Clin Nurs. diciembre de 2017;26(23-24):4537-47. | Population |
| Ying L, Ye Z, Xin Z, Junjun L, Li Z. Effectiveness of pelvic floor muscle biofeedback electrical stimulation combined with pelvic floor muscle training for mild to moderate postpartum stress urinary incontinence. Chinese J Perinat Med. 2023;26(3):230-5. | Non-English language |
| Zhu X-M; Jiang L-Q. (Effect of exercises and electrical stimulation of pelvic floor muscles on postpartum incontinence) [Chinese - simplified characters]. Nanfang Journal of Nursing 2012 Feb;19(2A):49-51. 2012; | Non-English language |

**Table S4.** Characteristics of the included studies.

| ***Randomized controlled trials studies*** | | | | | | | | | | | | | | | | | | | | | | | | | | | | | |  |  |  |  |  |
| --- | --- | --- | --- | --- | --- | --- | --- | --- | --- | --- | --- | --- | --- | --- | --- | --- | --- | --- | --- | --- | --- | --- | --- | --- | --- | --- | --- | --- | --- | --- | --- | --- | --- | --- |
| **Study characteristics** | | | | **Population characteristics** | | | | | | | | | | **Intervention characteristics** | | | | | | | | | | | | | **Outcome** | | | |  |  |  |  |
| **Reference** | | **Country** | | **Sample size** | | **Maternal age (years)** | | **Time after delivered (weeks)** | | **Delivery type (% vaginal)** | | **Primiparous (%)** | | **Intervention** | | | | | **Duration (weeks)** | | | **Frequency** | | | | **Questionnaire/objective measurement** | | | |  |  |  |  |  |
| Ahlund et al, 2013 | | Sweden | | IG:40  CG:42 | | IG:33  CG:33 | | 13 | | 100 | | 100 | | IG: instructions on how to contract PFM + short lecture (15 min) + written PFME program: 3 fast cc and 3 times 8–12 slow velocity, close to maximum cc (6 s).  CG: instructions on how to contract PFM | | | | 24 | | Daily | | | | | | | BFLUTS | | | | |  |  |  |
| Alahmri et al, 2024 | | Saudi Arabia | | IG1:13  IG2: 13 | | IG1:35  IG2:36.38 | | 0.25 | | 100 | | NR | | IG1: Home-based core exercises: basic bridge and static exercises for 10 s, 10 rep and relax; abdominal crunch exercises for 10 rep and relax (3 sets per day); PFMT: cc for 10 s, 3 s for relax, and repeated 3 sets (30 Min).  IG2: home-based PFMT: cc for 10 s, 3 s for relax, and repeated 3 sets (30 min) | | | | | 8 | | 3 times/week | | | | | | ICIQ-SF | | | | |  |  |  |
| Dai et al, 2024 | | China | | IG1:35  IG2:35 | | 27.89 | | 6 | | 100 | | 100 | | IG1: warm needle therapy (30 min) and perineal and anal cc of 3 s, followed by a period of relaxation of the same duration; 3 sets, and relax for 6 s (10 min).  IG2: same treatment without warm needle therapy. | | | | | 4 | | | 3 times/day | | | | ICIQ-SF | | | |  |  |  |  |  |
| Dumoulin et al, 2004 | | | Canada | | IG1:20  IG2:23  CG:19 | | IG1:36  IG2:37  CG:35.5 | | 12 | | NR | | NR | | IG1: ES: frequency 50 Hz, pulse width 250 µs, duty cycle 6 s on and 18 s off for first 4 weeks and 8 s on and 24 s off for last 4 weeks (15 min) + PFMT with BFB (25 min) + home exercise program  IG2: ES: frequency 50 Hz, pulse width 250 µs, duty cycle 6 s on and 18 s off for first 4 weeks and 8 s on and 24 s off for last 4 weeks (15 min) + PFMT with BFB (25 min) + home exercise program + AMT: isolation, re-education and retraining of trasversus abdominis (30 min).  CG: Relaxation massage for the back and extremities | | | 8 | | Weekly; Home exercise program = 5 times/ week | | | | | | | | | UDI | | | | |  |
| Glazener et al, 2001 | | | New  Zealand | | IG:371  CG:376 | | IG:29.6  CG:29.4 | | 48 | | 92 | | 36.7 | | IG: instructions on teaching PF anatomy + PFMT: 8-10 sessions per day, involving fast and slow cc, with the aim of 80-100 cc daily + bladder training.  CG: peripartum preparation | | | 28 | | | Daily | | Any pad use | | | | | | | | | | |  |
| Khorasani et al, 2020 | | | Iran | | IG:40  CG:40 | | IG:30.75  CG:30.25 | | 24 | | 100 | | NR | | IG: PFME at home (each set of 10 rep of 3 different types of exercise each week. Each cc involved 8–10 s hold time and the same rest time), performing correct transversus abdominis cc using BFB.  CG: No intervention | | | 12 | | | 3 times/week  (3 set a day) | | | | ICIQ-SF | | | | | | | | | |
| Kim et al, 2012 | | Korea | | IG1:9  IG2:9 | | 31.44 | | 6 | | 100 | | NR | | IG1: PFMT in various positions, abdominal strengthening exercises and trunk stabilization using a therapeutic ball (60 min)  IG2: same unsupervised treatment | | | | | 8 | | | 3 times/week | | | | BFLUTS | | | |  |  |  |  |  |
| Lange et al, 2024 | | Germany | | IG1:17  IG2:18  CG:17 | | 32.12 | | 9 | | 94.1 | | 47.1 | | IG1: PFMT was performed in individual courses by trained physiotherapists.  IG2: vaginal cube pessary individually adapted to each patient.  CG: standard care | | | | | 12 | | | NR | | | | self-reported satisfaction | | | |  |  |  |  |  |
| Liu et al, 2018 | | China | | IG1:50  IG2:50 | | IG1:50.35  IG2: 50.37 | | NR | | IG1:60  IG2:58 | | NR | | IG1: BFB-guided PFMT, ES adjusted to 8 to 32 Hz and 320 to 740 μs to induce contractions of the PFM to recover the cc and coordination of the abdominal and perineal muscles (30-40 min).  IG2: Sustained cc of the PFM for 3 to 5 s and relaxation for 3 s. Gradually increased series. | | | | | 8 | | | IG1:9 times/month  IG2: NR | | | | VTV (ml/72 h) | | | |  |  |  |  |  |
| Saboia et al, 2024 | | | Brazil | | IG:74  CG:64 | | IG:25  CG:27 | | 12 | | 100 | | 0 | | IG: Continence App®+PFMT  CG: standard care | | | 12 | | | at least once a day | | | | ICIQ-SF | | | | | | | | | |
| Shivkumar et al, 2015 | | | India | | IG:15  CG:15 | | 27.5 | | NR | | NR | | NR | | IG: Bladder training +slow cc of the PFM for 5 s. Same exercise for 10-50 s / 5 times  CG: bladder training | | | 8 | | | NR | | | | VAS | | | | | | | | | |
| Sigurdardottir et al, 2020 | | | Iceland | | IG:41  CG:43 | | IG:28  CG:29 | | 24 | | 90 | | 100 | | IG: verbal instructions to perform a correct PFM cc + PFMT with BFB:10 close-to-maximum cc and 7-s holding periods with a 10-s rest.  CG: verbal instructions about how to perform a correct PFM cc. | | | 12 | | | 3 times/day | | | | Australian pelvic floor Questionnaire | | | | | | | | | |
| Wang et al, 2023 | | China | | IG1:12  IG2:12  IG3:12 | | IG1:29.17  IG2:28.58  IG3:29.43 | | IG1:8  IG2:7.75  IG3:7.93 | | IG1:58.33  IG2:75  IG3:71.42 | | IG1:41.66  IG2: 66.66  IG3:50 | | IG1: explanation of PF anatomy and diet + guided PFMT (5 s of cc and 5 s of relax) through App (60 min).  IG2: explanation of PF anatomy and diet + guided AMT (Pilates) through App (60 min)  IG3: explanation of PF anatomy and diet + PFMT (5 s of cc and 5 s of relax) (60 min) | | | | | 8 | | | Daily | | | | ICIQ-SF | | | |  |  |  |  |  |
| Wang et al, 2024 | | China | | IG1:223  IG2:229 | | 34 | | 12.5 | | IG1:89.7  IG2: 87.3 | | 100 | | IG1: App + demonstration video and a booklet with explanations of the connection, operation, and maintenance of the home-based BFB device  IG2: App + PFMT programme. | | | | | 12 | | | 3 sets (6 min) each day | | | | ICIQ-SF | | | |  |  |  |  |  |
| Wilson et al, 1998 | | New Zealand | | IG1:19  IG2:21  IG3:14 | | 39.8 | | 48 | | 82 | | 28 | | IG1: PFMT: fast and slow cc, with the aim of 80-100 cc daily.  IG2: PFMT with cones: 9 cones in each set, increasing in weight from 20 to 100 g (15 min)  IG3: IG1+IG2 | | | | | 36 | | | IG1:8-10 times/day  IG2: 2 times/day  IG3: IG1+IG2 | | | | Pad test | | | |  |  |  |  |  |
| Zhou et al, 2020 | | China | | IG1:362  CG:358 | | 26.59 | | 7.28 | | 100 | | NR | | IG1: PFMT: voluntary cc and relax of the PFM at home (20 min). ES: frequency 50 Hz, stimulation cycle 2 s, and wave width 200 µS (15 min); after 10 min, intensity was increased to a level that did not cause noticeable discomfort. BFB: the patient was guided to actively carry out PFM cc and relaxation training (15 min).  CG: same treatment but after 10 min of ES the current intensity was increased. | | | | | 8 | | | PFMT: 2 times/day; ES+BFB: 2 times/week | | | | ICIQ-SF | | | |  |  |  |  |  |
| ***Non-randomized studies*** | | | | | | | | | | | | | | | | | | | | | | | | | | | | |  |  |  |  |  |  |
| **Study characteristics** | | | | **Population characteristics** | | | | | | | | | | **Intervention characteristics** | | | | | | | | | **Outcome** | | | | | |  |  |  |  |  |  |
| **Reference** | | **Country** | | **Sample size** | | **Maternal age (years)** | | **Time after delivered (weeks)** | | **Delivery type (% vaginal)** | | **Primiparous (%)** | | **Intervention** | | **Duration (weeks)** | | | | **Frequency** | | | **Questionnaire/objective measurement** | | | | | |  |  |  |  |  |  |
| Chen et al, 2023 | | China | | IG1:40 | | IG1:31.98 | | IG1:6.24 | | IG1:100 | | IG1:100 | | IG1: PFMT: inhale/ contract MSP for 6-8 s, exhale/relax. Gradually extend the duration of the exercises (30 min) | | IG1:12 | | | | | IG1:3 times/day | | | ICIQ-SF | | | | | |  |  |  |  |  |
|  | |  | | IG2:40 | | IG2: 32.01 | | IG2:6.52 | | IG2:100 | | IG2:100 | | IG2: ES: high frequency and high current intensity, not exceed 100 mA (30 min) | | IG2:12 | | | | | IG1:3 times/week | | | ICIQ-SF | | | | | |  |  |  |  |  |
|  | |  | | IG3:40 | | IG3: 31.89 | | IG3:6.16 | | IG3:100 | | IG3:100 | | IG3: PFMT: inhale/contract MSP for 6-8 s, exhale/relax. Gradually extend the duration of the exercises (30 min) + ES: high frequency and high current intensity, not exceed 100 mA (30 min) | | IG3:12 | | | | | IG3: PFMT: 3 times/day; ES:2 times/week | | | ICIQ-SF | | | | | |  |  |  |  |  |
| Juez et al, 2019 (A) | | Spain | | IG1:51 | | IG1:31.6 | | IG1:12 | | IG1:100 | | IG1:100 | | IG1: PFMT: 3 sets of 8 to 12 close to  maximum PFM cc per session, holding each cc for 6 to 8 s, in various positions (45-60 min) | | IG1:8 | | | | | IG1: On alternate days | | | ICIQ-SF | | | | | |  |  |  |  |  |
|  | |  | | IG2:54 | | IG2:32 | | IG2:12 | | IG2:100 | | IG2:100 | | IG2: AHT: 3 normal breathing cycles with slow diaphragmatic inspiration, a total air expiration and 25 s apnoea‐holding  after rib‐cage expansion, in various positions (45-60 min) | | IG2:8 | | | | | IG2: On alternate days | | | ICIQ-SF | | | | | |  |  |  |  |  |
| Lu et al, 2020 | | China | | IG1:90 | | IG1:29.9 | | IG1:NR | | IG1: 82.2 | | IG1:98.3 | | IG1:15-20 min of BFB combined with ES (frequency 20-100 Hz, wave width at 200-500 µs) | | IG1:12 | | | | | IG1:2 times/week | | | ICIQ-SF | | | | | |  |  |  |  |  |
|  | |  | | IG2:90 | | IG2:30.3 | | IG2:NR | | IG2:75.5 | | IG2:98.3 | | IG2: Continual cc of PFM for 3-5 s with a rest for 3-5 s (15 cycles/time) | | IG2:12 | | | | | IG2:2 times/day | | | ICIQ-SF | | | | | |  |  |  |  |  |

IG: Intervention Group; CG: Control Group; PF: Pelvic Floor; PFM: Pelvic Floor Muscle; PFME: Pelvic Floor Muscle Exercise; cc: contractions; PFMT: Pelvic Floor Muscle Training; ES: Electrical Stimulation; BFB: Biofeedback; AMT: Abdominal muscle training; NR: not reported; BFLUTS: Bristol Female Lower Urinary Tract Symptoms Questionnaire; UDI: Urogenital Distress Inventory; ICIQ-SF: International Consultation on Incontinence Questionnaire; VAS: Visual Analogue Scale; VTV: Volume Total Vesical; AHT: Abdominal hypopressive technique; EMG: Electromyography; Rep: Repetitions

**Table S5.** Subgroup analyses by:

|  |  |  | | |
| --- | --- | --- | --- | --- |
|  |  | n | SMD  (95% CI) | I^2^ |
| Maternal age | <30 years | 11 | -1.78  (-2.90, -0.66) | 97 |
|  | 30 to ≤35 years | 13 | -2.20  (-3.52, -0.87) | 96 |
|  | >35 years | 8 | -1.03  (-1,67, -0.38) | 57 |
| Time after delivery | ≤6 weeks | 7 | -1.21  (-2.51, 0.09) | 85 |
|  | >6 to ≤12 weeks | 14 | -1.89  (-3.09, -0.68) | 98 |
|  | >12 weeks | 7 | -1.79  (-2.76, -0.82) | 76 |
| Delivery type | Vaginal delivery | 15 | -2.08  (-3.25, -0.92) | 98 |
|  | Vaginal and no vaginal delivery | 15 | -1.23  (-1.71, -075) | 91 |
| Parity | Primiparous | 8 | -2.48  (-4.70, -0.27) | 97 |
|  | Primiparous and multiparous | 12 | -1.31  (-1.89, -0.73) | 93 |
|  | Multiparous | 1 | -0.42  (-0.66, -0.19) |  |

1. Characteristics of population
2. Characteristics of the intervention

| Length of the intervention |  |  | | |
| --- | --- | --- | --- | --- |
|  |  | n | SMD  (95% CI) | I^2^ |
|  | ≤8 weeks | 19 | -1.53  (-2.21, -0.84) | 96 |
|  | 12 weeks | 11 | -2.18  (-3.70, -0.65) | 97 |
|  | ≥24 weeks | 2 | -1.99  (-18.69, 14.71) | 91 |
|  | >40 sessions | 1 | -0.28  (-0.61, 0.06) |  |
| Number of sessions | <24 sessions | 9 | -1.68  (-2.43, -0.93) | 96 |
|  | ≤24 sessions | 2 | -1.09  (-8.52, 6.33) | 80 |
|  | 24 to ≤40 sessions | 11 | -1.33  (-2.41, -0.25) | 95 |
|  | >40 sessions | 8 | -2.43  (-4.37, -0.50) | 94 |

1. Type of outcome

|  |  | | |
| --- | --- | --- | --- |
|  | n | SMD  (95% CI) | I^2^ |
| Objective measurement | 6 | -1.74  (-2.95, -0.54) | 82 |
| Questionnaire | 27 | -1.74  (-2.48, -0.99) | 97 |

**Table S6.** Random effects meta-regression models

|  |  | | | |  |
| --- | --- | --- | --- | --- | --- |
|  | | **n** | **Coef.** | **p** | **R² (%)** |
| **Maternal age** | | 33 | 0.05 | 0.367 | 0 |
| **Time after delivery** | | 28 | -0.01 | 0.657 | 0 |
| **Length of the interventions** | | 30 | -0.06 | 0.384 | 0 |
| **Number of sessions** | | 26 | -0.01 | 0.016 | 18.87 |

**Figure S1.** Risk of bias of randomized controlled trials studies (ROB-2)

**Figure S2**. Risk of bias of non-randomized studies (ROBINS-I)

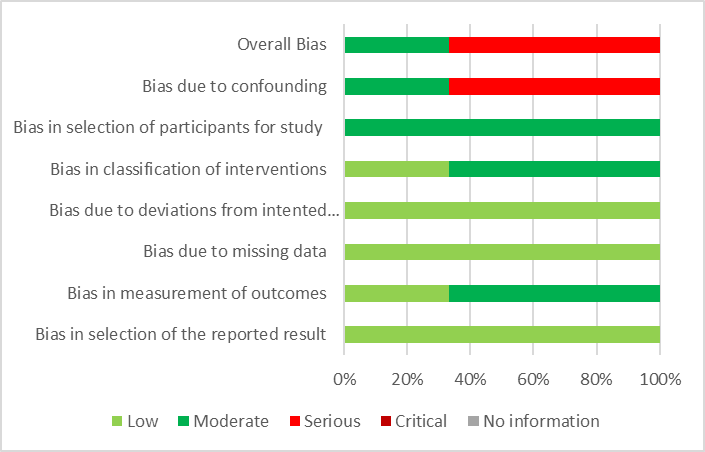


**Figure S3.** Sensitivity analysis.


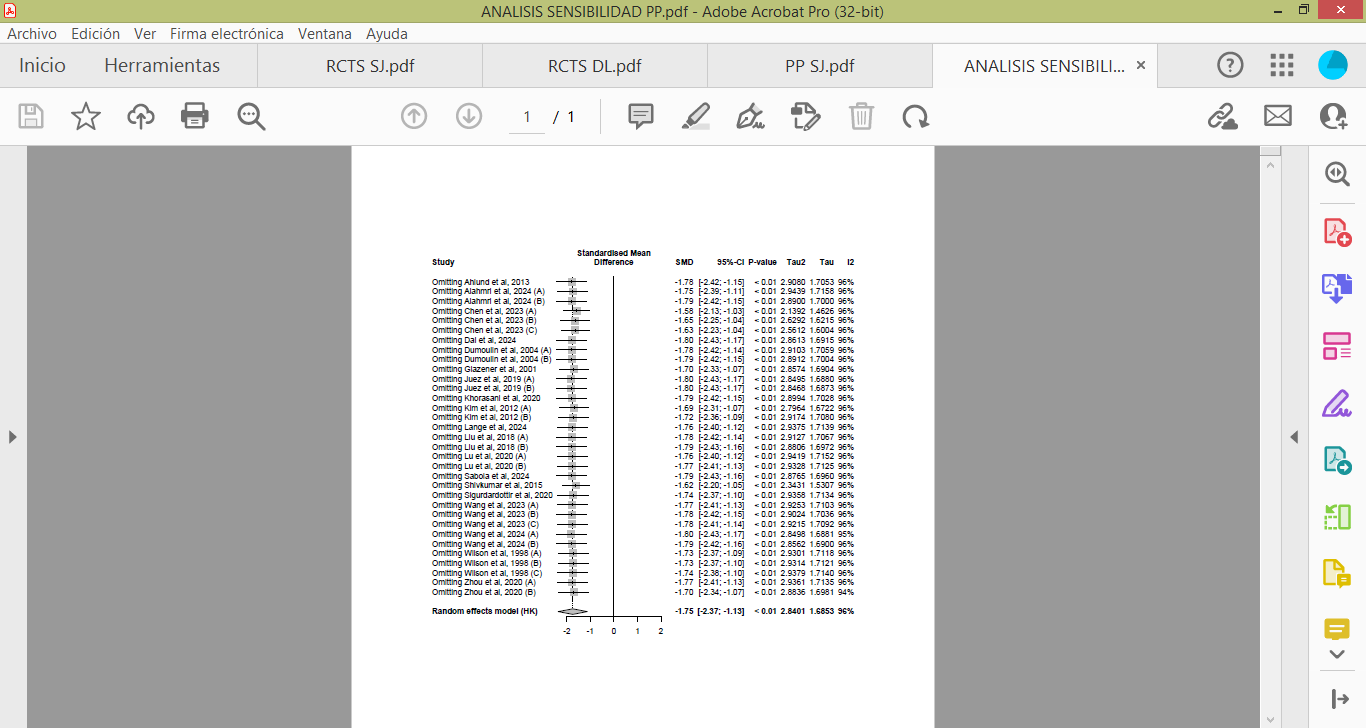


**Figure S4**. Forest plot showing the effect size of pre-post training interventions on UI during postpartum period using Der Simonian and Laird method.


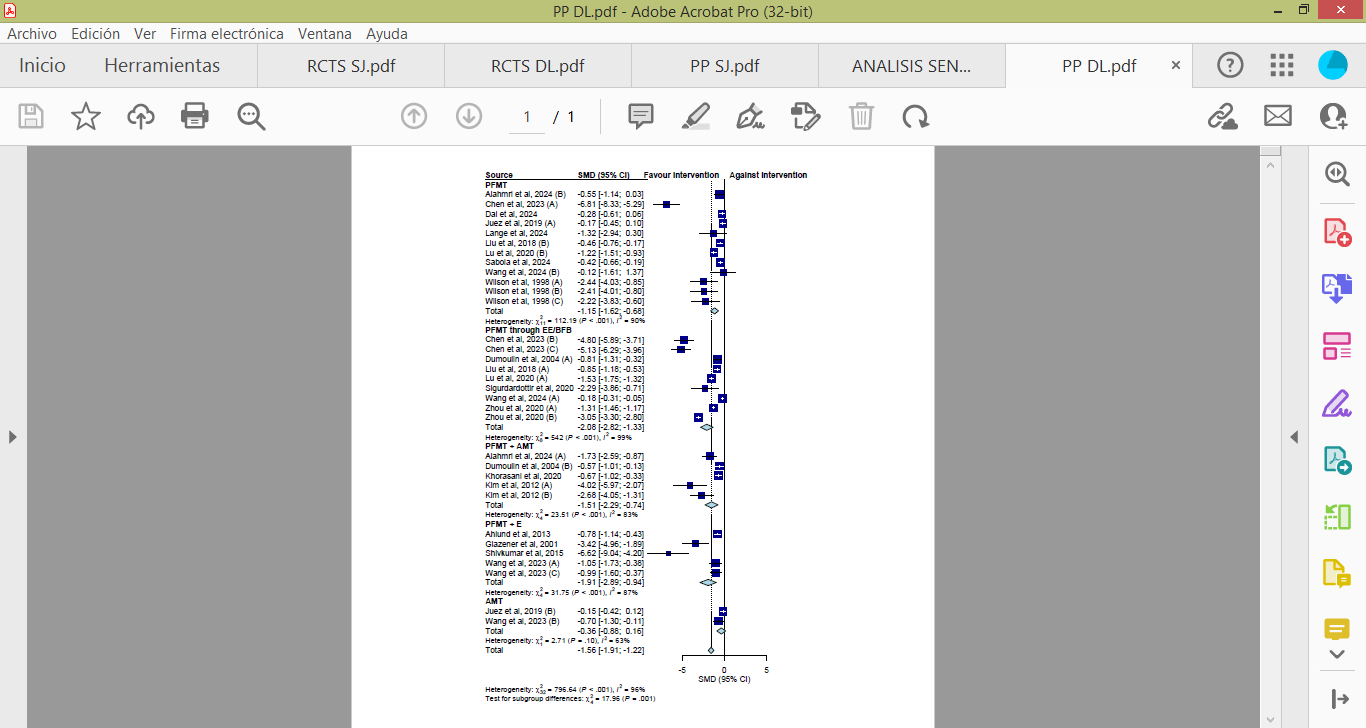


**Figure S5.** Forest plot of randomized controlled trials showing the effect size of. training interventions on UI during postpartum period versus education or control conditions using Der Simonian and Laird method.


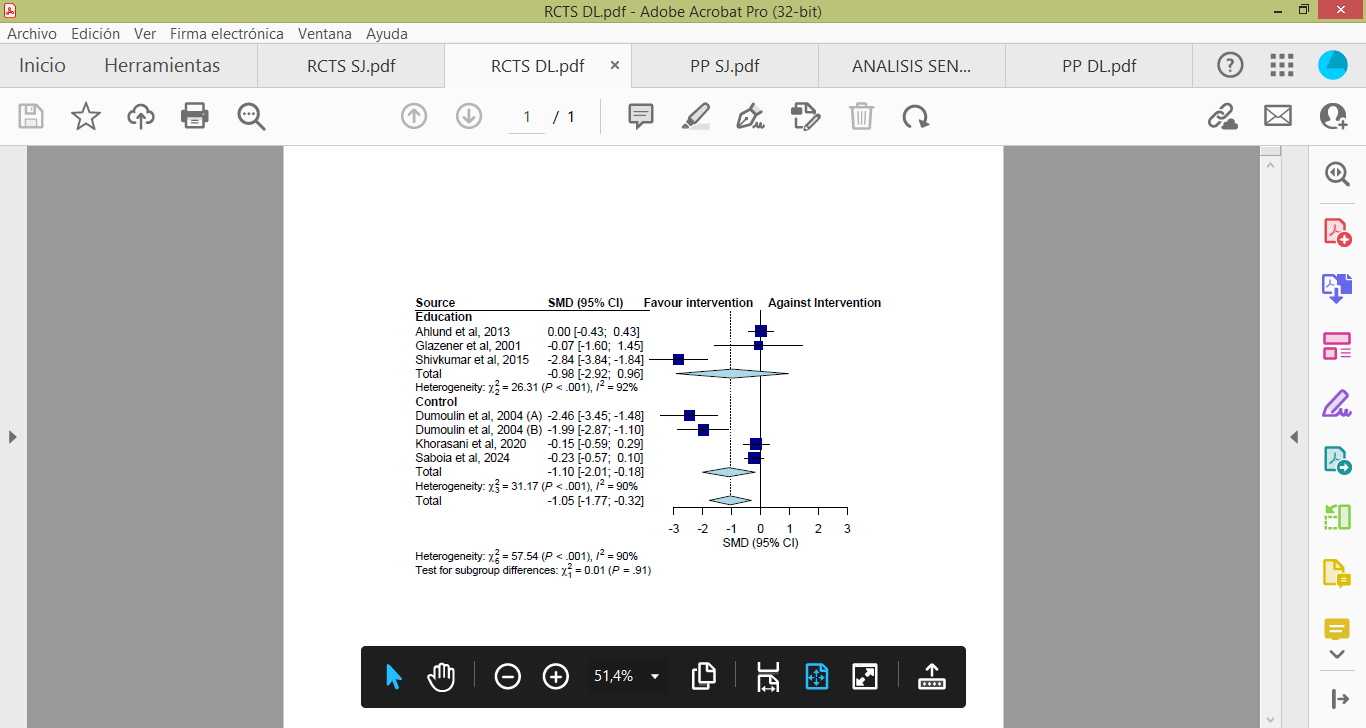


**Figure S6.** Funnel plot.


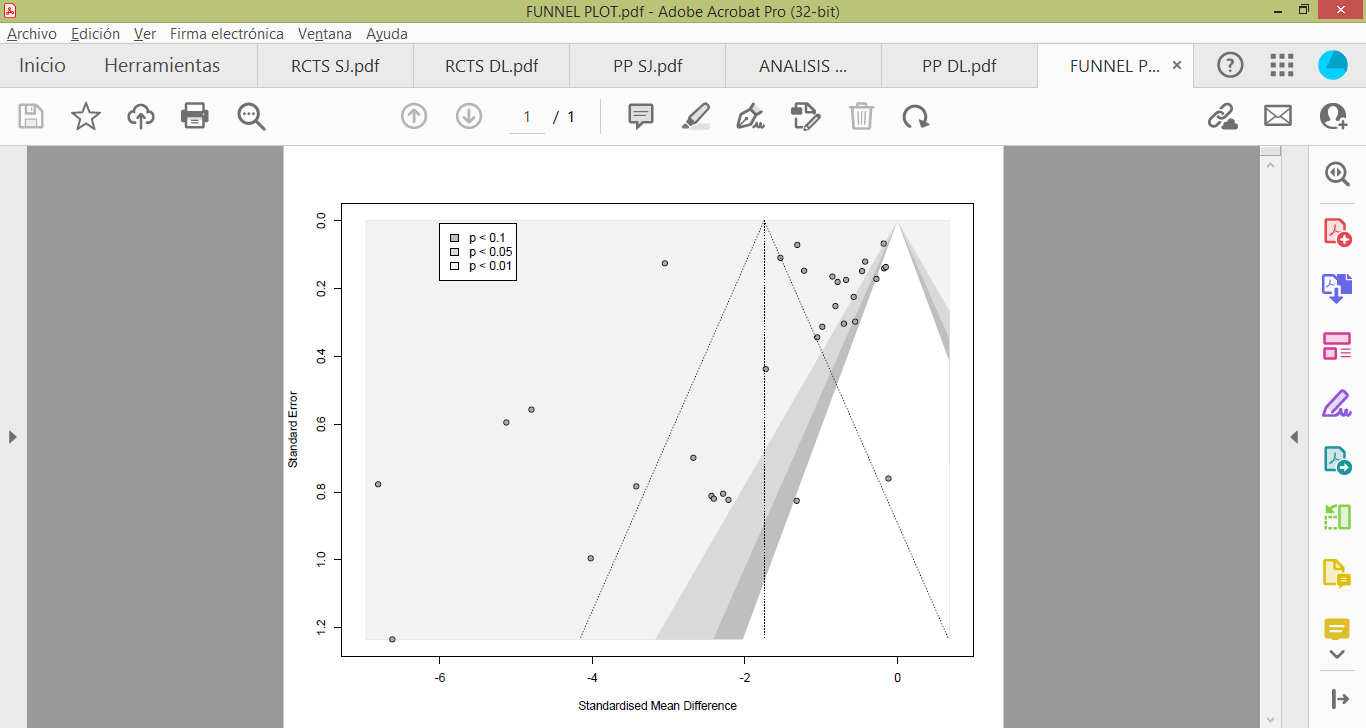

Supplement: Supplementary file 1 — Data S1: bjo70014‐sup‐0001‐DataS1.docx. [file BJO-133-243-s001.docx]
